# Overexpression in metastatic breast cancer supports Syndecan-1 as a marker of invasiveness and poor prognosis

**DOI:** 10.1007/s10238-022-00880-7

**Published:** 2022-09-10

**Authors:** Bruna Cerbelli, Annalinda Pisano, Maria Gemma Pignataro, Angelina Pernazza, Andrea Botticelli, Mariantonia Carosi, Leopoldo Costarelli, Matteo Allegretti, Giulia d’Amati, Iole Cordone

**Affiliations:** 1 Department of Medico-Surgical Sciences and Biotechnologies, Sapienza University, Latina, Italy; 2 Department of Radiological, Oncological and Pathological Sciences, Sapienza University, AOU Policlinico Umberto I, Viale Regina Elena 324, 00161 Rome, Italy; 3 Department of Clinical and Molecular Medicine, Sapienza University, Rome, Italy; 4 Department of Research, Advanced Diagnostics and Technological Innovation, IRCCS Regina Elena National Cancer Institute, Via E. Chianesi 53, 00144 Rome, Italy; 5 Department of Pathology, San Giovanni-Addolorata Hospital, Rome, Italy

**Keywords:** Syndecan-1 expression, Breast cancer, Brain metastases, Membrane localization, Metastatic process

## Abstract

**Background:**

Metastasis is the main cause of breast cancer (BC) mortality. Increasing evidence points to a role of syndecan-1 (CD138) expression as a prognostic marker involved in BC tissue and leptomeningeal metastasis. Aim of this study was to investigate and compare syndecan-1 tissue expression and localization in primary and secondary BC, focusing on brain metastases.

**Methods:**

Syndecan-1 expression was determined by immunohistochemistry. Focal *vs* diffuse (< or > 50% of cancer cells, respectively) pattern of expression, cellular localization (cytoplasm *vs* membrane) and intensity of immunostaining on neoplastic cells were evaluated. Moreover, the extent and pattern of expression of syndecan-1 were compared between primary tumors and paired metastases and correlated with the tumor intrinsic subtype.

**Results:**

A total of 23 cases, 10 with paired primary and metastatic tumor and 13 brain metastases, were evaluated. Syndecan-1 was expressed in both primary and metastatic BC. A diffuse cytoplasmic expression was observed in most primary BCs; by contrast, all metastatic lesions showed a membrane pattern of expression, suggesting a shift in cellular localization of syndecan-1 during the metastatic process. Concerning the extent of expression, we observed in metastatic lesions, a trend of association between intrinsic subtypes and extent of positivity. In particular, both BC characterized by overexpression of HER2 and triple-negative tumors were correlated with a diffuse pattern of expression with a moderate to strong intensity.

**Conclusion:**

A diffuse cytoplasmic expression was observed in most primary BCs; by contrast, all metastatic lesions showed a membrane pattern of expression, suggesting a shift in cellular localization of syndecan-1 during the metastatic process.

## Introduction

Breast cancer (BC) is the most common cancer in women, and the second leading cause of cancer death worldwide. Despite new therapies have significantly improved patient outcome, long-term survivors are facing an increased incidence of metastases. The pathogenic mechanisms underlying cancer development and spreading are extremely complex, and traditional BC classification, based on clinical-pathologic features and assessment of conventional biomarkers, does not capture the variability of the clinical courses in individual patients.

Central nervous system (CNS) metastasis accounts for up to 30% of the BC localizations after bone, liver and lung [1], and it is one of the most devastating complications with a very poor prognosis [2]. Higher incidence of brain metastasis has been reported in triple-negative (TN) and HER2 positive BC, although this complication is not exclusive of these aggressive subtypes [3]. Consequently, the importance and urgency of finding prognostic markers for CNS metastasis from BC and possible target molecules for efficient therapeutic strategies are a research topic of primary relevance.

Increasing evidence points to a possible role of syndecan-1 (CD138) expression as a prognostic marker involved in BC metastatic process [4]. Syndecan-1 is one of the four members of syndecan transmembrane heparan sulfate proteoglycans that are found in normal epithelial cells but also in different types of carcinomas, including BC [5]. In normal tissue, syndecan family members modulate several cellular processes such as adhesion, proliferation and migration, as well as cytoskeleton organization [6]. Possibly involved in brain metastatic process, specific patterns of CD138 immunohistochemical expression in neoplastic and stromal cells have been correlated with different clinical outcomes [5]. In addition, higher syndecan-1 mRNA levels have been positively correlated with HER2 amplification and negatively correlated with ER expression [7, 8]. Finally, silencing of syndecan-1 in BC experimental models reduces the rate of brain metastasis [9].

We have recently shown that surface syndecan-1 is over-expressed on cerebrospinal fluid (CSF) floating cancer cells of patients with BC leptomeningeal metastasis. In the same study, a strong syndecan-1 expression was documented in a subset of patients for which paired primary lesions were available [10]. We now aim (i) to compare syndecan-1 expression and cellular localization in primary and secondary BC, focusing on brain metastasis, to highlight possible differential expression patterns between primary and metastatic lesions; (ii) to verify the possible association of syndecan-1 expression pattern with BC intrinsic subtypes.

## Materials and methods

### Study approval and samples collection

This study was performed in line with the principles of the Declaration of Helsinki. Informed consent was obtained from all individual participants included in the study. A total of 23 cases, 10 with paired primary and secondary tumors (1 adrenal gland, 1 lung, 4 liver and 4 brain metastases) and 13 brain metastases for which the primary tumor was not available, were selected. Histologic features, tumor grade and intrinsic subtypes of all samples are listed in Table 1.

**Table 1:** Sample histology and intrinsic subtypes. CNS: central nervous system; NOS: not otherwise specified; NST: no special type

Patient IDs	Age at diagnosis (years)	Primary tumor			Metastasis		
		Histologic type	Tumor grade	Intrinsic subtype	Site	Tumor grade	Intrinsic subtype
1	32	NST	G2	Luminal A-like	Lung	G2	Luminal A-like
2	51	NST	G2	TN	Liver	G2	TN
3	70	Lobular	G2	Luminal B-like (HER2 positive)	Liver	G2	Luminal B-like (HER2 positive)
4	57	NST	G2	Luminal B-like (HER2 negative)	Liver	G1	Luminal B-like (HER2 negative)
5	59	NST	G3	Luminal B-like (HER2 negative)	Liver	G2	Luminal A-like
6	64	NST	G3	Luminal B-like (HER2 negative)	Adrenal	G2	Luminal B-like (HER2 negative)
7	53	NST	G3	Luminal B-like (HER2 negative)	CNS parietal lobe	G3	TN
8	52	NST	G3	HER2 positive	CNS temporal lobe	G3	HER2 positive
9	48	NST	G3	TN	CNS parietal lobe	G3	TN
10	49	NST	G2	Luminal B-like (HER2 negative)	CNS frontal lobe	G3	TN
11	47		Not available		CNS cerebellum	G3	Luminal B-like (HER2 negative)
12	70		Not available		CNS occipital lobe	G3	Luminal B-like (HER2 negative)
13	62		Not available		CNS cerebellum	G3	HER2 positive
14	70		Not available		CNS cerebellum	G3	TN
15	56		Not available		CNS frontal lobe	G2	Luminal A-like
16	52		Not available		CNS parietal lobe	G2	Luminal B-like (HER2 positive)
17	73		Not available		CNS cerebellum	G2	Luminal B-like (HER2 negative)
18	70		Not available		CNS occipital lobe	G2	Luminal B-like (HER2 negative)
19	51		Not available		CNS NOS	G2	Luminal B-like (HER2 positive)
20	50		Not available		CNS cerebellum	G3	Luminal B-like (HER2 negative)
21	70		Not available		CNS frontal	G2	Luminal B-like (HER2 negative)
22	47		Not available		CNS NOS	G3	HER2 positive
23	67		Not available		CNS NOS	G3	HER2 positive

### Immunohistochemical evaluation of syndecan-1 expression

**Fig. 1:**
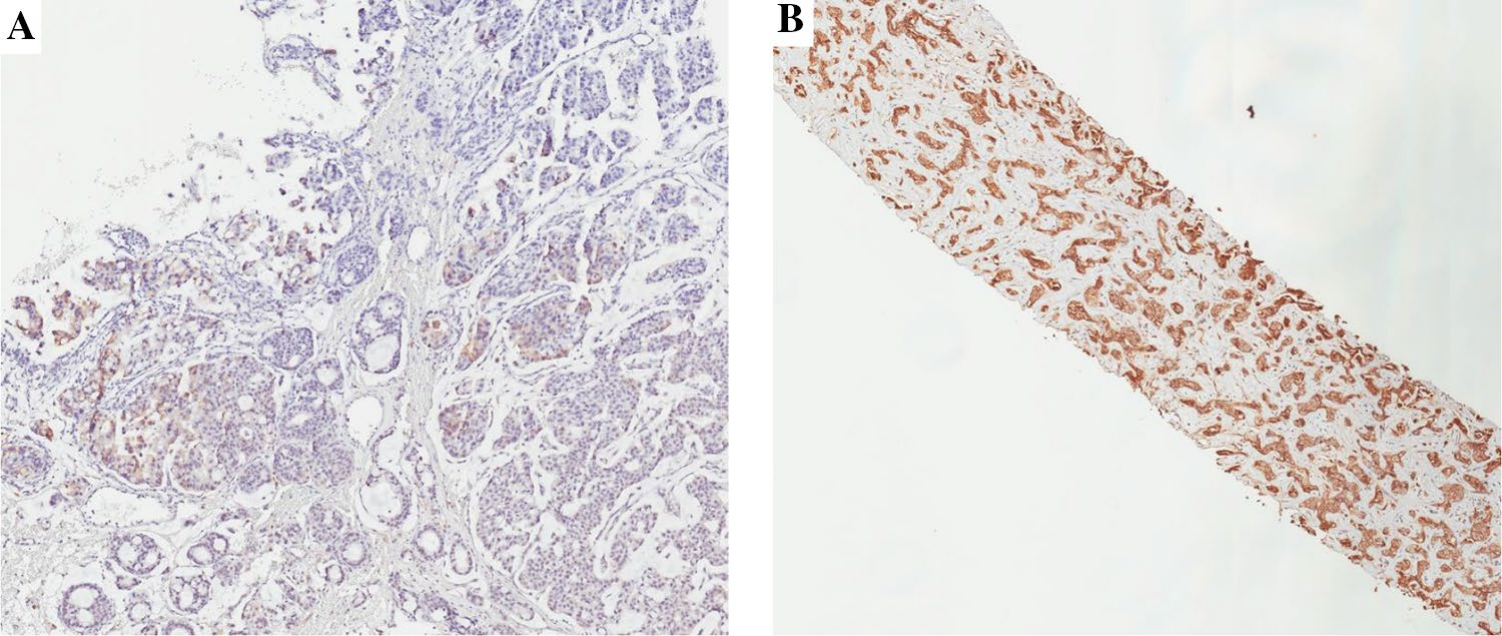
Evaluation of the extent of syndecan-1 on neoplastic cells. **A–B** Focal (< 50% of cells; **A**) and diffuse (≥ 50% of cells; **B)** extent of syndecan-1 expression (syndecan-1 immunohistochemical staining, original magnification × 5)

**Fig. 2:**
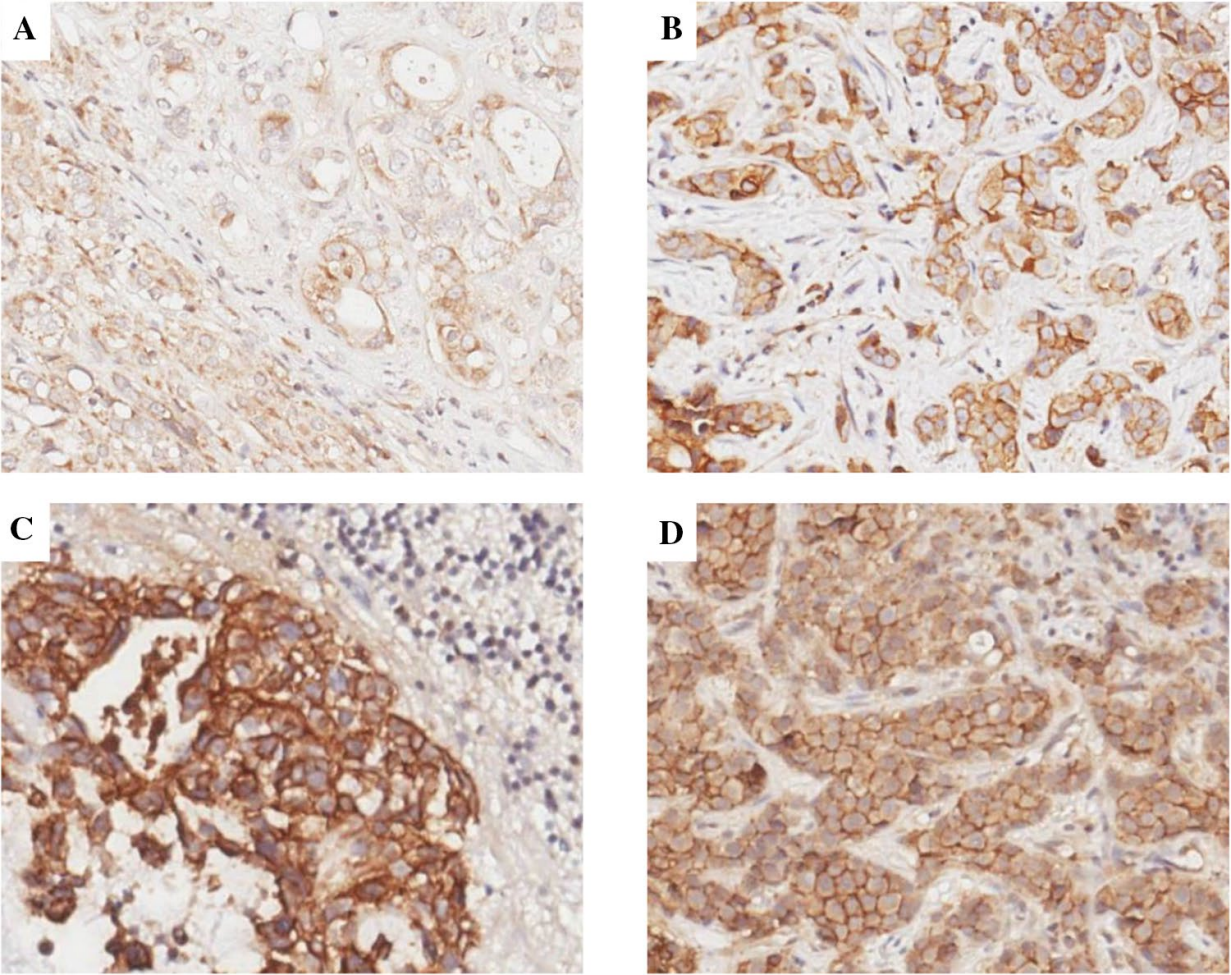
Pattern of expression of syndecan-1 in primary and metastatic breast cancer.** A–B** Cytoplasmic (**A**) and membranous (**B**) syndecan-1 stain in primary breast tumor (syndecan-1 immunohistochemical staining, original magnification × 20). **C–D** Membranous syndecan-1 stain in metastatic breast tumor (brain and liver metastasis **C–D**, respectively. Syndecan-1 immunohistochemical staining, original magnification × 20)

Serial sections were obtained from paraffin blocks representative of the surgical samples of both primary tumors and metastatic lesions for immunohistochemical evaluation of syndecan-1 expression. Immunohistochemistry was performed with standard streptavidin–biotin-peroxidase using a mouse IgG monoclonal antibody anti-CD138 (clone MI15, Dako, Glostrup, Denmark). Both positive and negative controls were stained in the same experiment. Immunohistochemical staining was evaluated on the whole sections by two observers (BC and GdA) according to the following parameters: i) the presence and extent of syndecan-1 expression on neoplastic cells (focal: < 50% of cells; diffuse: ≥ 50% of cells) (Fig. 1); (ii) cellular localization (cytoplasm *and/or* membrane) (Fig. 2); (iii) intensity of immunostaining on neoplastic cells expressed as a semiquantitative parameter (scored as low = 1 + , moderate = 2 + and strong = 3 +); (iv) immunostaining of stromal cells in the primary tumors, irrespective of extension and intensity. The expression pattern of syndecan-1 was then compared between primary tumor and paired metastases, and between the different metastatic sites, when available. Finally, the extent and pattern of expression of syndecan-1 on neoplastic cells were correlated with the intrinsic subtype of each tumor.

## Results

### Histological and immunohistochemical features of primary and metastatic BC

Matched primary and metastatic tumors were available from 10 patients who underwent surgery for BC between 2009 and 2017 and developed metastases that were resected between 2015 and 2020. The mean time elapsed between surgery for the primary tumor and resection of metastatic lesions was 52.3 months (range 24–132 months). Thirteen brain metastases without the corresponding primary tumor were also retrieved for the study. The histological features and intrinsic subtypes of the study cases, according to San Gallen classification [11], are detailed in Table 1. Among primary BC samples, 9 (90%) were of “no special type” (NST) and 1 was a lobular carcinoma. Fifty percent (5/10) were classified as Luminal B-like (HER2 negative), 2 cases (20%) were triple-negative (TN), 1 (10%) was Luminal A-like, 1 (10%) was Luminal B-like (HER2 positive) and 1 (10%) was HER2 positive. Regarding the tumor grade of primary BC, 50% were poorly differentiated (G3), while the remaining were moderately differentiated (G2); the degree of differentiation was the same in 6/10 paired primary and metastatic lesions. The intrinsic subtype was the same in 70% (7/10) of primary and paired metastatic lesion, while in 3 cases, there was a switch from the primary lesion (from Luminal B to TN in 2 cases and from Luminal B-like (HER2 negative) to Luminal A-like in 1 case).

Focusing on the 23 metastatic tumors, 17 were in the central nervous system (CNS), 4 in the liver, 1 in the lung and 1 in the adrenal gland (Table 1). CNS metastases were poorly differentiated in 11/17 (64.7%) cases and moderately differentiated in the remaining 6 cases; the most frequent intrinsic subtype of this subset of metastatic lesion was Luminal B-like (HER2 negative) (6/17, 35.3%), followed by HER2 positive (4/17, 23.5%), TN (4/17, 23.5%), Luminal B-like (HER2 positive) (2/17, 11.8%) and Luminal A-like (1/17, 5.9%).

### Pattern of syndecan-1 immunostaining in primary and metastatic BC

Localization, extent, and intensity of syndecan-1 immunostaining in primary and metastatic BC are summarized in Table 2. Representative pictures of syndecan-1 staining patterns in primary BC are provided in Fig. 2A–B.

**Table 2:** Localization, extent and intensity of syndecan-1 immunostaining in primary and metastatic breast cancer. Intensity of syndecan-1 immunostaining was scored as 1 + (low), 2 + (moderate) and 3 + (strong). CNS: central nervous system; F: focal, < 50% of cells; D: diffuse, ≥ 50% of cells

patient IDs	Primary tumor			Metastasis		
	Syndecan-1 extent/intensity on neoplastic cells		Stromal CD138 expression	Site	Syndecan-1 extent/intensity on neoplastic cells	
	Cytoplasmic	Membrane			Cytoplasmic	Membrane
1	D/2 +	F/1 +	neg	Lung	neg	F/1 +
2	neg	F/1 +	neg	Liver	F/1 +	F/1 +
3	D/2 +	D/2 +	neg	Liver	neg	D/2 +
4	neg	F/1 +	neg	Liver	F/1 +	D/2 +
5	D/2 +	F/1 +	neg	Liver	neg	D/2 +
6	F/1 +	D/2 +	neg	Adrenal	D/2 +	D/2 +
7	D/2 +	neg	pos	CNS	neg	D/2 +
8	F/1 +	neg	neg	CNS	neg	F/2 +
9	D/3 +	D/3 +	neg	CNS	neg	D/3 +
10	F/1 +	D/3 +	neg	CNS	F/1 +	D/3 +
11		Not available		CNS	neg	F/2 +
12		Not available		CNS	neg	F/2 +
13		Not available		CNS	F/1 +	D/3 +
14		Not available		CNS	neg	D/3 +
15		Not available		CNS	neg	F/2 +
16		Not available		CNS	neg	D/2 +
17		Not available		CNS	neg	F/1 +
18		Not available		CNS	neg	D/2 +
19		Not available		CNS	neg	D/1 +
20		Not available		CNS	neg	D/2 +
21		Not available		CNS	neg	F/1 +
22		Not available		CNS	neg	D/1 +
23		Not available		CNS	neg	D/2 +

Cytoplasmic expression of syndecan-1 was observed in most primary BCs (8/10, 80%). A diffuse pattern of staining with moderate/strong intensity was present in 5/8 cases (62.5%); the coexistence of cytoplasmic and membrane staining was a common finding (6/8, 75%). Immunostaining of stromal cells was observed only in 1 out of the 10 primary tumors. Normal breast epithelium showed weak syndecan-1 cytoplasmic staining.

Syndecan-1 expression was observed in all metastatic lesions, although it was decreased as compared to primary lesions and limited mostly to cell membranes. In fact, all metastatic lesions showed a membrane pattern of syndecan-1 expression, which was diffuse and with moderate/strong intensity in most of the samples (15/23, 65.2% and 17/23, 73.9%, respectively). At variance with primary tumors, coexistence of membrane and cytoplasmic expression was observed only in 5/23 cases (21.7%). Interestingly, CNS metastases showed the highest frequency of the exclusive membrane pattern of staining (15/17, 88%). Representative pictures of syndecan-1 staining patterns in different metastatic sites are provided in Fig. 2C–D.

Interestingly, when comparing each primary tumor with the corresponding metastatic lesion, we observed a decrease in cytoplasmic positivity of tumor cells (from 80 to 40% of cases).

Regarding the expression pattern of syndecan-1 in the different BC intrinsic subtypes, we did not observe a difference in the localization of this marker.

Concerning the extent of syndecan-1 expression, we observed, only in metastatic lesions, a trend of association between intrinsic subtypes and extent of positivity. In particular, we showed that TN (4/5, 80%) and tumors characterized by overexpression of HER2 (Luminal B-like (HER2 positive) 3/3, 100% and HER2 positive 3/4, 75%) were associated with a diffuse pattern of expression with a more prevalent moderate to strong intensity. However, a larger study population is needed to confirm this result.

## Discussion

Metastasis is the main cause of BC mortality, with a significant correlation with the intrinsic subtype of the primary tumor [3, 12]. Brain metastases represent one of the most devastating complications, with a very limited survival. Therefore, biomarkers able to identify patients at risk of metastatic spread are urgently needed to develop early detection methods and more effective treatment strategies.

Recent data have pointed to syndecan-1 as a potential marker of aggressive BC and adverse clinical outcomes [5, 7–9]. However, most of the data are related to syndecan-1 expression in primary breast cancer, with conflicting results possibly due to the patient’s selection criteria and the methods used. According to the tumor stem cell hypothesis, a subset of cells, defined as cancer-initiating cells, has a primary relevance in tumor metastases and cancer recurrence after chemotherapy [13]. A significant upregulation of syndecan-1 and a positive correlation with the expression of CD44, a marker of cancer stem cell (CSC) phenotype, have been documented in inflammatory breast cancer, a particularly aggressive BC subtype. Moreover, CSC phenotype was reduced upon syndecan-1 knockdown [14]. Similarly, syndecan-1 overexpression associated with stem cell markers phenotype has been documented in breast cancer leptomeningeal metastasis [10].

This paper focuses on syndecan-1 expression on primary breast cancers and metastatic lesions comparing, in a subset of cases, the pattern of CD138 immunohistochemical expression both in primary and paired metastatic tissue. Moreover, we focused our study on brain metastases (17/23 cases) based on our previous findings documenting surface syndecan-1 overexpression on CSF floating cancer cells of BC patients with central nervous system involvement [10].

We showed a different pattern of localization and immunostaining intensity between primary and metastatic lesions.

In fact, a diffuse cytoplasmic expression was observed in most primary BCs; by contrast, all metastatic lesions showed a membrane staining which was the exclusive pattern of expression in 15/17 cases (88%).

These findings suggest a shift in the cellular localization of syndecan-1 during the metastatic process, which could account for a role of this biomarker in favoring the escape from the primary site and the engraftment of neoplastic cells in the metastatic site. Our report expands previous observations pointing to the role of syndecan-1 in the complex reciprocal interaction between multiple myeloma cells and their bone marrow niche [15].

According to our results, syndecan-1 was expressed by stromal cells only in 1 out of 10 primary tumors (10%). This finding is in line with a previous report showing that syndecan-1 staining is expressed less frequently in stromal than in epithelial cells [4].

Finally, we did not observe a difference in the pattern of syndecan-1 expression according to BC intrinsic subtypes; however, metastatic lesions both TN and with HER2 overexpression (with or without hormone receptor expression) showed a more diffuse pattern of immunostaining, with moderate to strong intensity, compared to their Luminal A-like and Luminal B-like (HER2 negative) counterparts. Larger study samples are required to confirm this finding.

Nevertheless, we shown a direct association between specific intrinsic subtypes of BC and a higher metastatic propensity. TN and HER2 positive subtypes are commonly associated with aggressive phenotypes, higher rate of recurrences and a prevalence of brain metastasis.

Validation of our results in a larger scale and a comparison between primary and metastatic lesions are needed to confirm CD138 as a marker of metastatic potential and poor prognosis and a putative molecular target for innovative treatment strategies.

## Conclusions

Syndecan-1 is expressed in the neoplastic cells of both primary and metastatic BC, with a strikingly different cellular localization. However, metastatic lesions show a decreased expression of this marker with a selective membrane localization. These data expand previous observations in other epithelial tumors pointing to a possible role of syndecan-1 decrease in metastatic process. Moreover, we observed an association between the pattern of syndecan-1 expression and the BC intrinsic subtype of metastatic lesions, with a more diffuse pattern of immunostaining in TN and HER2 overexpressing lesions (with or without hormone receptor positivity). In line with the strong surface expression documented by flow cytometry in BC leptomeningeal metastasis, syndecan-1 overexpression has the potential to represent a reliable marker for anchorage-independent BC cells identification.

## Abbreviations

BC: Breast cancer
CNS: Central nervous system
TN: Triple-negative
CSF: Cerebrospinal fluid
NST: No special type
CSC: Cancer stem cell
